# Association between helicopter with physician versus ground emergency medical services and survival of adults with major trauma in Japan

**DOI:** 10.1186/cc13981

**Published:** 2014-07-09

**Authors:** Toshikazu Abe, Osamu Takahashi, Daizoh Saitoh, Yasuharu Tokuda

**Affiliations:** 1 Department of Emergency and Critical Care Medicine, Mito Kyodo General Hospital, University of Tsukuba, 3-2-7, Miyamachi, Mito, Ibaraki, Japan; 2 St Luke’s Life Science Institute Center for Clinical Epidemiology, 10-1, Akashicho, Chuo-ku, Tokyo 104-0044, Japan; 3 Department of Traumatology and Emergency Medicine, National Defense Medical College, 3-2, Namiki, Tokorozawa, Saitama 359-8513, Japan; 4 Japan Community Healthcare Organization, 3-22-12, Takanawa, Minato-ku, Tokyo 108-0074, Japan

## Abstract

**Introduction:**

Helicopter emergency medical services with a physician (HEMS) has been provided in Japan since 2001. However, HEMS and its possible effect on outcomes for severe trauma patients have still been debated as helicopter services require expensive and limited resources. Our aim was to analyze the association between the use of helicopters with a physician versus ground services and survival among adults with serious traumatic injuries.

**Methods:**

This multicenter prospective observational study involved 24,293 patients. All patients were older than 15 years of age, had sustained blunt or penetrating trauma and had an Injury Severity Score (ISS) higher than 15. All of the patient data were recorded between 2004 and 2011 in the Japan Trauma Data Bank, which includes data from 114 major emergency hospitals in Japan. The primary outcome was survival to discharge from hospitals. The intervention was either transport by helicopter with a physician or ground emergency services.

**Results:**

A total of 2,090 patients in the sample were transported by helicopter, and 22,203 were transported by ground. Overall, 546 patients (26.1%) transported by helicopter died compared to 5,765 patients (26.0%) transported by ground emergency services. Patients transported by helicopter had higher ISSs than those transported by ground. In multivariable logistic regression, helicopter transport had an odds ratio (OR) for survival to discharge of 1.277 (95% confidence interval (CI), 1.049 to 1.556) after adjusting for age, sex, mechanism of injury, type of trauma, initial vital signs (including systolic blood pressure, heart rate and respiratory rate), ISS and prehospital treatment (including intubation, airway protection maneuver and intravenous fluid). In the propensity score–matched cohort, helicopter transport was associated with improved odds of survival compared to ground transport (OR, 1.446; 95% CI, 1.220 to 1.714). In conditional logistic regression, after adjusting for prehospital treatment (including intubation, airway protection maneuver and intravenous fluid), similar positive associations were observed (OR, 1.230; 95% CI, 1.017 to 1.488).

**Conclusions:**

Among patients with major trauma in Japan, transport by helicopter with a physician may be associated with improved survival to hospital discharge compared to ground emergency services after controlling for multiple known confounders.

## Introduction

Helicopters have been used to transport trauma patients for the past the 40 years all over the world [1]. Physician-staffed helicopter emergency medical services (HEMS) started in Japan in 2001. HEMS and their possible effect on outcomes for severe trauma patients have been debated. Researchers in the United States [2] and Germany [1] have shown the benefits of HEMS. The results of other studies show no significant benefits [3,4]. Actually, helicopter use seems to provide benefits for trauma patients. It can reduce rescue time and enlarge the catchment area. However, the utility of HEMS depends on time and weather. Also, HEMS cost much more than ground emergency medical services (GEMS). Thus, clear evidence of their effectiveness compared with GEMS is worth seeking because HEMS require limited and expensive resources. Also, knowledge about the effectiveness of HEMS in Japan is important because it is a mountainous island country smaller than the United States. Thus, our purpose in the present study was to analyze and validate the association between the use of HEMS versus GEMS and survival among adults with serious traumatic injuries.

## Methods

### Ethics statement

We received permission to use data from the Japan Trauma Data Bank (JTDB). The study protocol was reviewed and approved by the ethics committee of Mito Kyodo General Hospital, University of Tsukuba Hospital Mito Medical Center. The ethics committee at our institution does not require consent from patients for observational studies using anonymous data such as those used in this study.

### Study design and data collection

This multicenter, prospective, observational study was devoted to analysis of the association between the use of HEMS versus GEMS and survival among adults with serious traumatic injuries. The data used in this study were derived from the JTDB, which was established in 2003 with the Japanese Association for the Surgery of Trauma (Trauma Registry Committee) and the Japanese Association for Acute Medicine (Committee for Clinical Care Evaluation) as the main parties. The aim of establishing the JTDB was to collect and analyze trauma patient data in Japan (patient and injury characteristics, information from emergency services, vital signs before reaching the hospital and at the first medical examination, inspections and treatments, diagnosis and Injury Severity Score (ISS), disposition after being in the ED or the operating room and information upon discharge from the hospital). The severity of anatomic injuries was evaluated using the ISS. Probability of survival was also calculated on the basis of the Trauma Score and Injury Severity Score [5].

### Selection of participants

**Figure 1:**
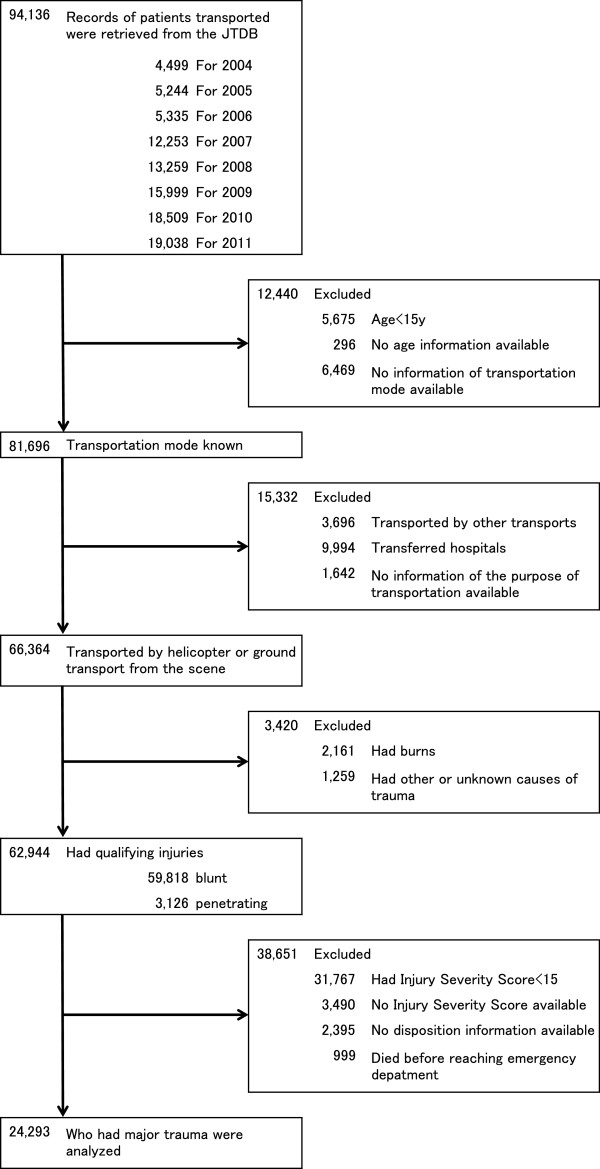
**Study flow diagram.** Stratification and selection of patient data retrieved from the Japan Trauma Data Bank (JTDB) covering the period from 2004 to 2011.

A total of 94,136 patients were enrolled in the JTDB from 2004 to 2011. Our study included adults older than 15 years of age. The ISS was used to quantify the severity of trauma. An ISS higher than 15 was used as the criterion for inclusion because this has been shown to be associated with a greater need for specialized trauma care [2]. Patients who received other forms of transportation to trauma centers, such as private conveyance or other purposes of transfer to hospitals, were also excluded. Patients with other trauma mechanisms, such as burns, were excluded. Patients who were dead on arrival to the emergency department (ED) were excluded. The analysis was restricted to records with complete information regarding transport and disposition information. Thus, 24,293 patients met our study criteria (Figure 1).

### Statistical analysis

#### ***Data included in the analysis***

The primary intervention was transport by either HEMS or GEMS. The dispatch criteria for HEMS in Japan included shock, long-distance transportation of critical patients, swift transportation due to requirement of special care, such as for burns, polytrauma, amputated extremity or need for emergency care by a physician at the scene. However, the number of HEMS did not cover all such patients. Thus, it depended on the decisions of the operators. The HEMS team was staffed by a physician and a nurse. The GEMS team was staffed by an emergency medical technician and a firefighter. The primary outcome of interest was survival to discharge from the hospital. The secondary outcome of interest was survival to discharge from the ED. These outcomes were evaluated with three analytical models: an unconditional (standard) logistic regression model, a logistic regression model incorporating the results of propensity score matching and a conditional logistic regression model.

Covariates included demographic, physiologic and hospital data. Demographics included information on age and sex. Referring to the previous report [2], covariates were carefully selected on the basis of the assumption that none were affected directly by the intervention. Other variables selected *a priori* were planned for inclusion in the final statistical models. These variables included the type of trauma (blunt versus penetrating), vital signs at the scene (systolic blood pressure (SBP), respiratory rate (RR), heart rate (HR)) and the locally calculated ISS. ISS and sex were also found to be statistically significant covariates in a previous study in which the investigators compared urban and rural helicopter transport of patients with blunt trauma injuries [6]. The final models included the following independent variables: age; sex; initial vital signs, including SBP, RR and HR; mechanism of injury; type of trauma; transport mode; ISS; and prehospital treatment, including intubation, airway protection maneuver and intravenous fluid. The two-sided significance level for all tests was *P* < 0.05. All analyses were performed using SPSS software, version 21.0 (IBM, Armonk, NY, USA).

#### ***Propensity score***

The use of helicopter or ground transport was not randomly assigned, and thus, as the described in the previous report [2], formal causal inference is not credible. Therefore, we used a propensity score approach to condition potential selection bias and confounding. The propensity score model was estimated using a logistic regression model to adjust for the patient characteristics chosen in Table 1. We carefully chose these variables. The variables were significant variables between the two groups. Also, we chose these variables because they had fewer missing values. We referred to prognostically significant variables in the previous study [2]. Age, sex, mechanism of injury, type of trauma, initial vital signs (SBP, HR, RR) and ISS were chosen as the covariates used to estimate the propensity score. This propensity score represents the probability that a patient with severe trauma would be chosen for transport by helicopter or ground. Next, we chose one-to-one matching, which is the most common procedure. One patient transported by helicopter was matched to one patient transported by ground using nearest-neighbor matching without replacement. Propensity scores were matched using a caliper width of 0.25 (standard deviation of the logit). Also, we used a structured iterative approach to refine this logistic regression model to achieve balance of covariates within the matched pairs. We used the standardized difference to measure covariate balance, whereby an absolute standardized difference above 20% represents meaningful imbalance. Paired comparisons were performed using conditional logistic regression analysis because propensity-matched models still have imbalance. For conditional logistic regression analysis, we adjusted prehospital treatment, including intubation, airway protection maneuver and intravenous fluid, because there was significant imbalance between the two groups.

**Table 1:** **Characteristics of patients transported by emergency medical services**^**a**^

		**Patients by transportation method, *n *** **(%)**	
	**All patients**	**Helicopter**	**Ground**
**Characteristics**	**(*N*** **= 24,293)**	**(*N*** **= 2,090)**	**(*N*** **= 22,203)**
	*n* (%)		
Sex			
Men	17,265 (71.1)	1,571 (75.2)	15,694 (70.7)
Women	7,023 (28.9)	519 (24.8)	6,504 (29.3)
Age, yr			
15 to 55	11,749 (48.4)	884 (42.3)	10,865 (48.9)
56 to 65	4,279 (17.6)	419 (20.0)	3,860 917.4)
>65	8,265 (34.0)	787 (37.7)	7,478 (33.7)
Injury type			
Blunt	23,680 (97.5)	2,062 (98.7)	21,618 (97.4)
Penetrating	613 (2.5)	585 (2.6)	28 (1.3)
Cause of injury			
Motor vehicle crash	12,638 (53.9)	1,087 (53.2)	11,551 (54.0)
Falls	8,749 (37.3)	675 (33.0)	8,074 (37.7)
Other reason	2,059 (8.8)	283 (13.8)	1,776 (8.3)
Injury severity score			
15 to 24	11,747 (52.9)	892 (47.3)	10,855 (53.4)
25 to 34	8,184 (36.8)	738 (39.1)	7,446 (36.6)
35-44	2,279 910.3)	256 (13.6)	2,023 (10.0)
TRISS (P > 0.5)	16,578 (77.7)	1,435 (76.9)	15,143 (77.8)
Alcohol drunk (+)	2,876 (11.8)	53 (4.7)	2,823 (21.6)
	Mean (SD)		
Prehospital vital signs			
Systolic blood pressure, mmHg	132.3 (34.6)	130 (34.8)	132.5 (34.6)
Diastolic blood pressure, mmHg	77.7 (21.3)	76.7 (21.3)	77.8 (21.3)
Respiratory rate, breaths/min	23.0 (7.5)	24.9 (8.5)	22.8 (7.4)
Heart rate, beats/min	87.7 (22.6)	87.7 (23.8)	87.7 (22.5)
Japan coma scale			
Grade 0	5,077 (23.5)	408 (26.8)	4,669 (23.3)
Grade 1	6,635 (30.7)	421 (27.6)	6,214 (30.9)
Grade 2	2,591 (12.0)	209 (13.7)	2382 (11.8)
Grade 3	7,333 (33.9)	487 (31.9)	6846 (34.0)
Initial vital signs at ED			
Systolic blood pressure, mmHg	118.8 (51.6)	119.1 (44.0)	118.8 (52.2)
Diastolic blood pressure, mmHg	73.0 (27.2)	72.6 (23.8)	73.0 (27.5)
Respiratory rate, breaths/min	20.8 (9.9)	22.2 (9.5)	20.6 (9.9)
Heart rate, beats/min	82.1 (33.2)	85.9 (29.7)	81.8 (33.5)
Glasgow coma scale score	10.9 (4.6)	10.9 (4.7)	10.9 (4.6)
Eye score	2.8 (1.3)	2.8 (1.3)	2.8 (1.3)
Verbal score	3.4 (1.7)	3.7 (1.6)	3.4 (1.7)
Motor score	4.7 (1.9)	4.7 (2.0)	4.7 (1.9)
Japan coma scale			
Grade 0	5,191 (26.5)	540 (30.1)	4,651 (26.1)
Grade 1	5,127 (26.1)	407 (22.7)	4,720 (26.5)
Grade 2	2,990 (15.2)	260 (14.5)	2,730 (15.3)
Grade 3	6,304 (32.1)	588 (32.8)	5,716 (32.1)
Body temperature, °C	36.0 (1.1)	36.0 (1.1)	36.0 (1.1)

^a^ED: Emergency department; TRISS: Trauma Score and Injury Severity Score.

Japan coma scale (Grade 0: alert, Grade 1: possible eye-opening, not lucid, Grade 2: possible eye-opening upon stimulation, Grade 3: no eye-opening and coma).

Also, Japan coma scale is categorical data. Thus, its unit is n (%).

## Results

There were 24,293 patients in the JTDB who had major trauma, among whom 2,090 were transported by HEMS and 22,203 were transported by GEMS. Patient demographics and characteristics are summarized in Tables 1, 2 and 3. The mean age was 56.2 ± 19.6 in the HEMS group and 53.1 ± 21.1 in the GEMS cohort (*P* < 0.001). Patients transported by HEMS had higher ISSs (28.4 ± 12.7) than those transported by GEMS (26.8 ± 12.3) (*P* < 0.001). Among the patients who died, 546 (26.1%) were transported by HEMS and 5,765 patients (26.0%) by GEMS.

**Table 2:** **Characteristics of preexisting medical conditions**^**a**^

		**Patients by transportation method, *n *** **(%)**	
	**All patients, *n *** **(%)**	**Helicopter**	**Ground**
	**(*N*** **= 24,293)**	**(*N*** **= 2,090)**	**(*N*** **= 22,203)**
Neurological diseases			
Psychotic disorders	1,620 (6.7)	71 (3.4)	1,549 (7.0)
Dementia or mental retardation	479 (2.0)	21 (1.0)	458 (2.1)
Lung diseases			
Bronchial asthma	564 (2.3)	55 (2.6)	509 (2.3)
COPD	101 (0.4)	9 (0.4)	92 (0.4)
Cardiovascular diseases			
Stroke	779 (3.2)	59 (2.8)	720 (3.2)
Coronary heart disease	551 (2.3)	48 (2.3)	503 (2.3)
Congestive heart failure	221 (0.9)	12 (0.6)	209 (0.9)
Hypertension	3,310 (13.6)	294 (14.1)	3,016 (13.6)
Digestive diseases			
Peptic ulcer	428 (1.8)	40 (1.9)	388 (1.7)
Chronic hepatitis	314 (1.3)	19 (0.9)	295 (1.3)
Cirrhosis	183 (0.8)	13 (0.6)	170 (0.8)
IBD	106 (0.4)	16 (0.8)	90 (0.4)
Systemic diseases or diseases of other organs			
Diabetes	1,666 (6.9)	132 (6.3)	1,534 (6.9)
Dialysis	180 (0.7)	13 (0.6)	167 (0.8)
Active cancer	314 (1.3)	34 (1.6)	280 (1.3)
Marked obesity	38 (0.2)	4 (0.2)	34 (0.2)
Hematologic disorders	43 (0.2)	4 (0.2)	39 (0.2)
HIV infection	13 (0.1)	0 (0.0)	13 (0.1)
Pregnancy	15 (0.1)	1 (0.0)	14 (0.1)
Medications			
Anticoagulant	194 (0.8)	16 (0.8)	178 (0.8)
Steroid	48 (0.2)	3 (0.1)	45 (0.2)
Immunosuppressant	14 (0.1)	2 (0.1)	12 (0.1)
Previously healthy	1,4696 (60.5)	13,406 (60.4)	1,290 (61.7)

^a^COPD: Chronic obstructive pulmonary disease; IBD: Inflammatory bowel disease.

**Table 3:** **Characteristics of treatment and outcome among patients transported by emergency medical services**^**a**^

		**Patients by transportation method, *n *** **(%)**	
	**All patients, n (%)**	**Helicopter**	**Ground**
	**(*N*** **= 24,293)**	**(*N*** **= 2,090)**	**(*N*** **= 22,203)**
Prehospital treatment			
O_2_	17,110 (70.4)	1,475 (70.6)	15,635 (70.4)
Cervical collar	14,611 (60.1)	1,259 (60.2)	13,352 (60.1)
Backboard	14,181 (58.4)	1,349 (64.5)	12,832 (57.8)
Shock pants	21 (0.1)	2 (0.1)	19 (0.1)
Splint	309 (1.3)	22 (1.1)	287 (1.3)
Intubation	1,840 (7.6)	121 (5.8)	1,719 (7.7)
Nasal airway tube	240 (1.0)	10 (0.5)	230 (1.0)
Airway protection maneuver	2,023 (8.3)	233 (11.1)	1,790 (8.1)
Intravenous fluid	963 (4.0)	395 (18.9)	568 (2.6)
FAST at ED			
Positive	2,919 (12.7)	309 (15.4)	2,610 (12.4)
Negative	16,598 (71.9)	1,559 (77.8)	15,039 (71.4)
Not used	3,557 (15.4)	136 (6.8)	3,421 (16.2)
CT			
Head	20,473 (84.3)	1,708 (81.7)	18,765 (84.5)
Neck	12,246 (50.4)	1,138 (54.4)	11,108 (50.0)
Spine	4,845 (19.9)	485 (23.2)	4,360 (19.6)
Chest	16,155 (66.5)	1,577 (75.5)	14,578 (65.7)
Abdomen	15,376 (63.3)	1,502 (71.9)	13,874 (62.5)
Pelvis	12,424 (51.1)	1,006 (48.1)	11,418 (51.4)
Others	899 (3.7)	72 (3.4)	827 (3.7)
Angiography			
Head	298 (1.2)	27 (1.3)	271 (1.2)
Neck	131 (0.5)	12 (0.6)	119 (0.5)
Spine	46 (0.2)	4 (0.2)	42 (0.2)
Chest	335 (1.4)	36 (1.7)	299 (1.3)
Abdomen	1,190 (4.9)	119 (5.7)	1,071 (4.8)
Pelvis	1,215 (5.0)	143 (6.8)	1,072 (4.8)
Others	81 (0.3)	12 (0.6)	69 (0.3)
Blood transfusion (+)	6,611 (28.0)	782 (38.2)	5,829 (27.0)
Operation			
Craniotomy	2,055 (8.5)	142 (6.8)	1,913 (8.6)
Craterization	716 (2.9)	36 (1.7)	680 (3.1)
Throracotomy	947 (3.9)	130 (6.2)	817 (3.7)
Celiotomy	1,427 (5.9)	177 (8.5)	1,250 (5.6)
Bone fixation	3,606 (14.8)	432 (20.7)	3,174 (14.3)
Angiostomy	68 (0.3)	6 (0.3)	62 (0.3)
TAE	1,237 (5.1)	127 (6.1)	1,110 (5.0)
Endoscopic surgery	23 (0.1)	3 (0.1)	20 (0.1)
Anastomosis	9 (0.0)	1 (0.0)	8 (0.0)
Others	959 (3.9)	93 (4.4)	866 (3.9)
Disposition at ED			
Died	2,004 (8.2)	1,877 (8.5)	127 (6.1)
ICU	18,439 (75.9)	1,634 (78.2)	16,805 (75.7)
Ward	3,197 (13.2)	184 (8.8)	3,013 (13.6)
Others	452 (1.9)	139 (6.7)	313 (1.4)
Disposition at discharge			
Died	6,311 (26.0)	546 (26.1)	5,765 (26.0)
Hospital transfer	10,172 (41.9)	1,063 (50.9)	9,109 (41.0)
Home	7,810 (32.1)	481 (23.0)	7,329 (33.0)

^a^CT: Computed tomography; ED: Emergency department, FAST: Focused assessment with sonography in trauma; TAE: Transcatheter arterial embolization.

The results of the logistic regression models are listed in Table 4. Crude survival did not differ significantly between the HEMS and GEMS cohorts. However, HEMS showed a significantly greater odds ratio (OR) of survival in the standard logistic regression models (OR, 1.277 (95% confidence interval (CI), 1.049 to 1.556)).

**Table 4:** **Association between transportation method and survival outcome**^**a**^

	**Survival OR (95% CI)**	
**Analysis**	**At discharge**	**At ED**
Crude (*N* = 24,293)	0.992 (0.895 to 1.098)	1.437 (1.193 to 1.730)
Unconditional logistic regression		
Standard (*n* = 15,382)	1.277 (1.049 to 1.556)	1.141 (0.729 to 1.785)
After propensity score matching^b^ (*n* = 2,510)	1.446 (1.220 to 1.714)	2.367 (1.822 to 3.075)
Conditional logistic regression (*n* = 2,510)	1.230 (1.017 to 1.488)	1.703 (1.255 to 2.311)

^a^CI: Confidence interval; ED: Emergency department; OR: Odds ratio. ^b^The covariates used to estimate the propensity score were age, sex, mechanism of injury, type of trauma, initial vital signs (systolic blood pressure, heart rate, risk ratio) and Injury Severity Score.

In addition, we analyzed the relationship between transport and survival in propensity-matched patients. To calculate the propensity score, a multivariable logistic regression model was fitted. This model yielded an area under the curve of 0.641 (95% CI, 0.625 to 0.658), which indicated a relatively good ability to differentiate those transported by HEMS from those by GEMS. To assess the robustness of the results, we performed a series of sensitivity analyses (Table 5). With respect to every predictor variable, significant differences in prehospital treatment were detected between patients transported by HEMS compared to GEMS. Thus, we used conditional logistic regression for prehospital treatment to be well-matched. The survival rates of patients transported by HEMS were significantly greater than those transported by GEMS (OR, 1.446 (95% CI, 1.220 to 1.714) after propensity score matching; OR, 1.230 (95% CI, 1.017 to 1.488) in conditional logistic regression) (Table 4).

**Table 5:** Baseline characteristics patients with severe trauma in propensity-matched patients

	**Patients by transportation method, *n *** **(%)**		
	**Helicopter**	**Ground**	
	**(*N*** **= 1,255)**	**(*N*** **= 1,255)**	**SD**^**a **^**(%)**
Males	949 (75.6)	918 (73.3)	5.3%
Age, yr	55.2 (19.5)	54.4 (20.8)	4.0%
Injury type			
Blunt	1,239 (98.7)	1,240 (98.8)	0.9%
Penetrating	16 (1.3)	15 (1.2)	
Cause of injury			
Motor vehicle crashes	656 (54.2)	613 (54.1)	9.8%
Falls	415 (34.3)	450 (39.7)	11.2%
Other reasons	139 (11.5)	70 (6.2)	18.7%
Injury severity score	27.5 (12.6)	28.3 (14.4)	5.9%
Prehospital vital signs			
Systolic blood pressure, mmHg	131.6 (86.9)	133.8 (36.2)	3.3%
Respiratory rate, breaths/min	23.5 (5.5)	22.8 (5.5)	12.7%
Heart rate, beats/min	86.9 (23.9)	87.0 (20.9)	0.4%
Prehospital treatment			
Intubation	70 (5.6)	164 (13.1)	26.0%
Airway protection maneuver	124 (9.9)	133 (10.6)	1.6%
Intravenous fluid	217 (17.3)	38 (3.0)	48.7%

^a^SD: Standardized difference.

## Discussion

Although the impact of HEMS and GEMS is still debated, our findings support the results of recent studies on HEMS with advanced statistics [1,2,7-10]. Actually, the ORs reported in those studies were very similar (Table 6). Our summary evaluation of these studies is that HEMS has a survival benefit compared to GEMS, although HEMS patients had more serious medical conditions. We added robust results in this area because we used rigorous methods after controlling for the same multiple known confounders that Galvagno *et al*. did [2]. However, because a helicopter is not a treatment, but instead just a vehicle, its effects include heterogeneity. It could be a surrogate marker. Also important is carefully consideration of which elements of HEMS may be beneficial for patients [11]. Other factors to consider are speed, distance, time, trauma center access, crew expertise, level of primary hospital treatment, duration of flight and severity of patient condition.

**Table 6:** **Summary of recent studies using multivariate logistic regression to compare helicopter versus ground emergency medical services**^**a**^

**Study**	**Number of patients**	**OR (95% CI) for HEMS survival**
Thomas *et al*. [7]		
HEMS	2,292	1.32 (1.03 to 1.71)
GEMS	14,407	
Brown *et al*. [8]		1.22 (1.18 to 1.27)
HEMS	41,987	
GEMS	216,400	
Stewart *et al*. [9]		
HEMS	2,739	1.49 (1.19 to 1.89)
GEMS	6,473	
Sullivant *et al*. [10]		
HEMS	10,049	1.64 (1.45 to 1.87)
GEMS	46,695	
Galvagno *et al*. [2]		
HEMS	47,637	1.16 (1.14 to 1.17)
GEMS	111,874	
Andruszkow *et al*. [1]		
HEMS	4,989	1.33 (1.16 to 1.57)
GEMS	8,231	
Present study (2014)		
HEMS	2,090	1.23 (1.02 to 1.49)
GEMS	22,203	

^a^CI: Confidence interval; GEMS: Ground emergency medical services; HEMS: Helicopter emergency medical services; OR: Odds ratio. Modification of Table 1 in Galvagno *et al*. [11].

Compared to GEMS, HEMS can transport patients with serious injuries greater distances significantly faster. Time to definitive trauma care (particularly hemostasis requiring surgery) is known to strongly influence outcome [12]. In a previous study, researchers suggested a road distance more than 10 miles or an expected transport time more than 45 minutes by GEMS is the minimum at which helicopter transport could be faster [13]. We do not have distance and time information in this database. However, we know the duration of transportation in many cases was less than these suggested thresholds, because there are many emergency centers in small areas in Japan. So, HEMS could have other merits besides access, including speed and distance.

Crew expertise is likely to be an important contributing factor for improved survival in HEMS patients. HEMS in Germany and Europe are exclusively physician-staffed, as in Japan. This could be a potential benefit. In the present study, prehospital treatment, especially intravenous fluid, was statistically significantly different because GEMS in Japan do not allow prehospital treatment, except for cardiopulmonary arrest patients. Being staffed by well-trained physicians must be a beneficial element. Being well-trained could be more important than physician-staffed, however, because, for example, many HEMS are not physician-staffed in the United States.

Variation in hospital capability has a potential impact on outcome. Biewener *et al*. concluded in their study that the level of primary hospital treatment, but not the transportation mode, influenced patients’ survival [3]. However, Andruszkow *et al*. reported that HEMS seemed to influence survival independently of level I treatment [1]. We did not control for the level of institutions; however, institutions that join JTDB meet a certain standard of trauma care, because almost all are emergency centers certified by the Japanese Ministry of Health, Labour and Welfare.

Another consideration is that HEMS operate only during daytime in Japan. That factor could affect outcome data, because hospital functions are more sophisticated in daytime than at night. However, HEMS also operate during the night in other countries. Moreover, with regard to the severity of patient condition, the authors of a previous report stated that patients transported by urban GEMS arrive at the hospital with more severe injuries and have higher in-hospital death rates than patients from rural areas who survive and are transported by HEMS [14]. However, we controlled for the severity of patients’ injuries by using the ISS as the researchers in a previous study did [2].

Our study has several limitations. First, the absence of distance and time information is one. Second, we did not control for staffing in each vehicle. Indeed, this difference is one of the most important confounders in this study. However, it is difficult to avoid this factor in the Japanese emergency system. An important factor related to staffing is prehospital treatment, and we controlled for intubation, airway protection maneuver and intravenous fluid. Third, we could not analyze the cost of each vehicle, because we did not obtain cost data. Nonetheless, public broadcasters in Japan indicate that emergency helicopters force Japanese communities to shoulder a huge financial burden. Fourth, we have missing data, which might have influenced the results. Although Moore *et al*. recommended using multiple imputation as a more accurate data model than the one we used [15], we chose the simple approach of eliminating all patients with missing data on important covariates because of the size of our data set. Actually, our data set had much less missing data than Galvagno *et al*.’s [2]. Fifth, there could be unknown bias, although we controlled for known bias by using a propensity score. However, our method is relatively robust because a randomized controlled trial comparing HEMS with GEMS is not feasible. Moreover, we did not evaluate the quality of posttrauma life, because we dealt only with the outcome of survival.

## Conclusion

The results of this study demonstrate that transport by helicopter with a physician may be associated with improved survival to hospital discharge compared to ground services, after controlling for multiple known confounders among patients with major trauma in Japan. Probably the combination of elements of HEMS has benefits for patients with trauma, although we could not detect the most likely contributing factor. Additional work is needed to determine whether transport by helicopter or time to high-level care, such as physician staffing, might be the association determinant of trauma survival.

## Key messages

• We add robust results to previously published data on the relative benefits of HEMS compared to GEMS based on our rigorous analytical methods after controlling for multiple known confounders.

• HEMS demonstrated a survival benefit compared to GEMS, although HEMS patients had more serious medical conditions.

## Abbreviations

ED: Emergency department; GEMS: Ground emergency medical services; HEMS: Helicopter emergency medical services; HR: Heart rate; ISS: Injury Severity Score; JTDB: Japan Trauma Data Bank; RR: Respiratory rate; SBP: Systolic blood pressure.

## Competing interests

The authors declare that they have no competing interests.

## Authors’ contributions

DS contributed to the acquisition of data. All of the authors jointly conceived of and designed this study. TA conducted data cleaning. TA and OT analyzed the data. All of the authors interpreted the data. TA drafted the manuscript. All of the authors reviewed and discussed the manuscript. TA, DS and YT revised the manuscript for important intellectual content. All of the authors read and approved the final manuscript.

## References

[B1] Andruszkow H Lefering R Frink M Mommsen P Zeckey C Rahe K Krettek C Hildebrand F Survival benefit of helicopter emergency medical services compared to ground emergency medical services in traumatized patients Crit Care 2013 17 R124 10.1186/cc12796 23799905 PMC4056624

[B2] Galvagno SM Jr Haut ER Zafar SN Millin MG Efron DT Koenig GJ Jr Baker SP Bowman SM Pronovost PJ Haider AH Association between helicopter vs ground emergency medical services and survival for adults with major trauma JAMA 2012 307 1602 1610 10.1001/jama.2012.467 22511688 PMC3684156

[B3] Biewener A Aschenbrenner U Rammelt S Grass R Zwipp H Impact of helicopter transport and hospital level on mortality of polytrauma patients J Trauma 2004 56 94 98 10.1097/01.TA.0000061883.92194.50 14749573

[B4] Bulger EM Guffey D Guyette FX MacDonald RD Brasel K Kerby JD Minei JP Warden C Rizoli S Morrison LJ Nichol G Resuscitation Outcomes Consortium Investigators Impact of prehospital mode of transport after severe injury: a multicenter evaluation from the Resuscitation Outcomes Consortium J Trauma Acute Care Surg 2012 72 567 575 quiz 803 10.1097/TA.0b013e31824baddf 22491538 PMC3495608

[B5] Boyd CR Tolson MA Copes WS Evaluating trauma care: the TRISS method. Trauma Score and the Injury Severity Score J Trauma 1987 27 370 378 10.1097/00005373-198704000-00005 3106646

[B6] McCowan CL Swanson ER Thomas F Handrahan DL Outcomes of blunt trauma victims transported by HEMS from rural and urban scenes Prehosp Emerg Care 2007 11 383 388 10.1080/10903120701536867 17907020

[B7] Thomas SH Cheema F Wedel SK Thomson D Trauma helicopter emergency medical services transport: annotated review of selected outcomes-related literature Prehosp Emerg Care 2002 6 359 371 10.1080/10903120290938508 12109585

[B8] Brown JB Stassen NA Bankey PE Sangosanya AT Cheng JD Gestring ML Helicopters and the civilian trauma system: national utilization patterns demonstrate improved outcomes after traumatic injury J Trauma 2010 69 1030 1036 10.1097/TA.0b013e3181f6f450 21068607

[B9] Stewart KE Cowan LD Thompson DM Sacra JC Albrecht R Association of direct helicopter versus ground transport and in-hospital mortality in trauma patients: a propensity score analysis Acad Emerg Med 2011 18 1208 1216 10.1111/j.1553-2712.2011.01207.x 22092906

[B10] Sullivent EE Faul M Wald MM Reduced mortality in injured adults transported by helicopter emergency medical services Prehosp Emerg Care 2011 15 295 302 10.3109/10903127.2011.569849 21524205

[B11] Galvagno SM Jr Comparative effectiveness of helicopter emergency medical services compared to ground emergency medical services Crit Care 2013 17 169 10.1186/cc12779 23890322 PMC4057392

[B12] McMonagle MP Flabouris A Parr MJ Sugrue M Reducing time to urgent surgery by transporting resources to the trauma patient ANZ J Surg 2007 77 241 246 10.1111/j.1445-2197.2007.04026.x 17388826

[B13] Diaz MA Hendey GW Bivins HG When is the helicopter faster? A comparison of helicopter and ground ambulance transport times J Trauma 2005 58 148 153 10.1097/01.TA.0000124264.43941.41 15674165

[B14] Rogers FB Shackford SR Hoyt DB Camp L Osler TM Mackersie RC Davis JW Trauma deaths in a mature urban vs rural trauma system: a comparison Arch Surg 1997 132 376 382 10.1001/archsurg.1997.01430280050007 9108758

[B15] Moore L Hanley JA Turgeon AF Lavoie A Émond M A multiple imputation model for imputing missing physiologic data in the National Trauma Data Bank J Am Coll Surg 2009 209 572 579 10.1016/j.jamcollsurg.2009.07.004 19854396

